# Foam pads properties and their effects on posturography in participants of different weight

**DOI:** 10.1186/s12998-014-0045-4

**Published:** 2015-01-19

**Authors:** Guy Gosselin, Michael Fagan

**Affiliations:** School of Engineering, University of Hull, Cottingham Road, Kingston-upon-Hull, HU6 7RX UK

**Keywords:** Balance, Foam pads, Posturography, Modulus of elasticity, Biomechanics

## Abstract

**Background:**

Foam pads are increasingly used on force platforms during balance assessments in order to produce increased instability thereby permitting the measurement of enhanced posturographic parameters. A variety of foam pads providing different material properties have thus been used, although it is still unclear which characteristics produce the most effective and reliable tests. Furthermore, the effects of participant bodyweight on the performance of the foam pads and outcome of the test are unknown. This project investigated how different foam samples affected postural sway velocity in participants of different weights.

**Method:**

Four foam types were tested according to a modified American Society for Testing and Materials standard method for testing flexible cellular materials. Thirty-six healthy male factory workers divided into three groups according to body mass were tested three times for each of the 13 randomly-selected experimental situations for changes in postural sway velocity in this cross-over study. Descriptive and inferential statistics were used to compare the results and evaluate the difference in sway velocity between mass groups.

**Results:**

For the materials considered here, the modulus of elasticity of the foam pads when compressed by 25% of their original heights was inversely proportional to their density. The largest changes in postural sway velocity were measured when the pads of highest stiffness were used, with memory foam pads being the least likely to produce significant changes.

**Conclusions:**

The type of foam pads used in posturography is indeed important. Our study shows that the samples with a higher modulus of elasticity produced the largest change in postural sway velocity during quiet stance. The results suggest that foam pads used for static computerised posturography should 1) possess a higher modulus of elasticity and 2) show linear deformation properties matched to the participants’ weight.

## Background

Posturography has been shown to be useful in the workplace, for example to assess different-aged workers in physically demanding jobs [1], determine the effects of obesity on balance [2], to measure sleepiness and fatigue [3], and even to observe the effects of neurotoxicity due to workers exposure to organic solvent mixtures [4]. Furthermore, there is now a trend in western countries to increase the age of retirement [5]. This aging workforce may in some instances be placed at risk should their functional capacities such as balance become altered. Approximately one person in three over the age of 65 has at least one fall a year and one person in five who falls after the age of 65 for reasons connected with balance dies in the year following the fall [6]. In addition obese adults fall nearly twice as often as their non-obese counterparts [7]. All this motivates researchers and clinicians to develop new ways to understand and quantify postural stability.

Postural stability is often assessed by measuring the centre of pressure (COP) which is a point where the vertical reaction forces of the ground act. It represents the weighted average of all pressures over the body in contact with the ground. As such, there are numerous COP measures such as average velocity of COP, COP excursion, average radial displacement of the COP, to name a few; however until recently it was not evident which measure is optimal [8]. Mahdavi-Amiri et al. have shown that during static posturography the average velocity for a given stability condition, is more repeatable (less variable) between trials from a data collection session, and more discernible between the different stability conditions [9].

Many of the modern assessments systems use dynamic posturographic devices, which are sophisticated apparatus that introduce instability along with altered visual cues [10]. Unfortunately the high costs of such systems together with their large size prevent their general use in industry. Static computerised posturography represents a low-cost alternative, although the current high variability of results limits the accuracy of the conclusions that can be drawn from such assessments [11].

Recently, foam pads have been used on force platforms in order to induce increased instability thereby decreasing the coefficient of variation (CV) to a more acceptable level [12]. The use of foam pads in posturography is thought to exagerate balance deficits by altering the reliability of somatosensory input from cutaneous mechanoreceptors on the plantar soles. Previous research looking at the effect of the surface on which posturography is performed has shown that the type of foam has different effects on balance [11,13]. Although it is still unclear which characteristics produce the optimal performance, De Berardino and colleagues suggested that using foam pads of higher stiffness was best for clinical use [11]. More specific information is therefore essential before a standardised protocol can be proposed. Foam pads used in posturography will behave as any other material when placed under load, i.e. the deflection will be proportional to both the force, by a property known as the stiffness of the structure, and proportional to the property of the material itself called the modulus of elasticity [14].

Few papers have reported the material characteristics of foam pads used in posturography. Blackburn (2003) [15] investigated the kinematic analysis of the hip and trunk during bilateral stance on firm foam and multiaxial support surfaces. In this instance the height of the foam blocks was not mentioned but the density was reported as 54.53 kg/m^3^ [15]. Another study that looked into trunk sway measurements during stance and gait tasks in Parkinson’s disease [16], used foam pads with a height of 10 cm and a density of 25 kg/m^3^. Finally, Di Bernardino et al. [11] evaluated the postural effects of standing on two different types of rubber foam pads: a “monolayer” with a thickness of 10 cm and a density of 25 kg/m^3^, and a “bilayer” pad with a thickness of 8 cm and a density of 100 kg/m^3^. Their results show that the variability of static posturography parameters was significantly reduced by the use of both foam pads. However, the comparison of the two types was also statistically significant, with the bilayer type presenting the lowest CV in the results of 10%, compared with 14.4% for the monolayer. Unfortunately, the bi-layer foam pad described by Di Berardino is a specialist product that it is not readily available outside of Italy.

To the best of our knowledge no one has investigated the postural effects of participants of different mass and the effects of plantar surface area on different types of foam. One would assume that the postural effects of standing on a specific foam pad sample would be different for lighter and heavier participants. Thus, this study’s main purpose was to determine how a range of foam pads (including bi-layer foam pad combinations) influenced postural sway velocity during quiet stance for subjects of different body mass. The null hypothesis tested was: there is no difference in sway velocity when any of the foam pads are used.

## Method

### Foam pads material properties

**Figure 1:**
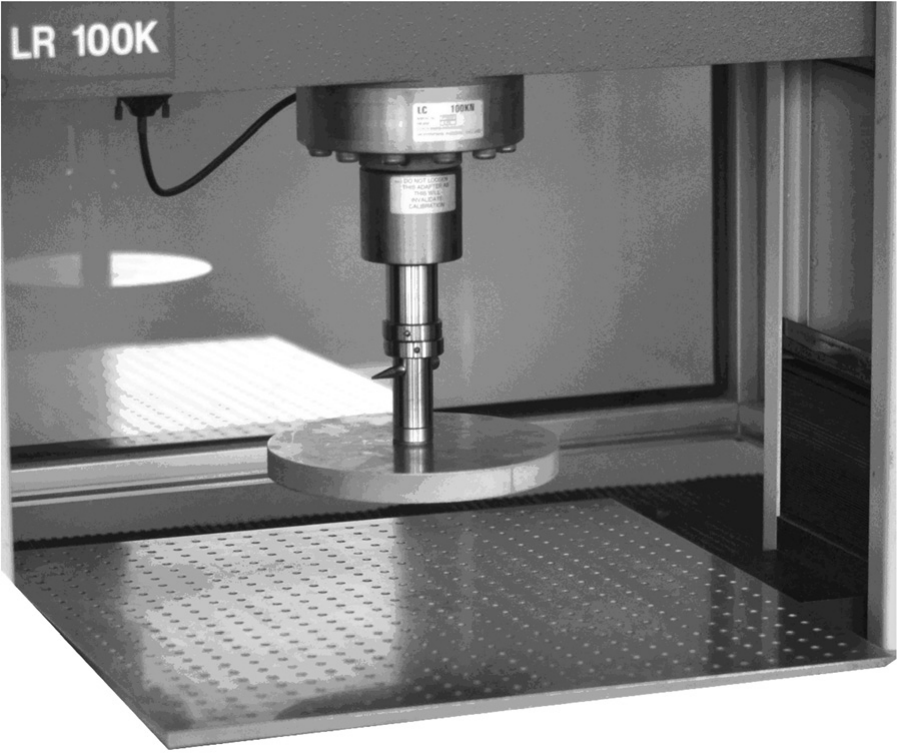
**Screw driven test machine (LR 100 K, Lloyds Instrument, Bognor Regis, UK) with a 100 kN load cell showing the 203 mm indenter foot above the perforated horizontal support plate.**

The properties of the pads were measured using three tests based on ASTM test D-3574-11 [17]. Uniaxial compression was achieved using a screw driven test machine (LR 100 K, Lloyds Instrument, Bognor Regis, UK) with a 100 kN load cell (Figure 1). The press was remotely controlled via a desktop computer running Nexygen software (Lloyds Instrument, Bognor Regis, UK). Four foam pads were obtained from three sources: 1) rehabilitation material supplier, 2) online foam shop and 3) upholstery high street shop (Table 1). The pads had a size of 480 × 480 mm, with the exception of the rehabilitation balance pad which had a smaller size of 440 × 400 mm. The atmospheric pressure in the laboratory was 1015 hPa and the temperature was 22°C.

**Table 1:** **Foam sample specification**

**Manufacturer**	**Model**	**Type**	**Size**	**Volume**	**Mass**	**Density**	***E***
			**(mm)**	**(m** ^**3**^ **)**	**(kg)**	**kg/m** ^**3**^	**kPa**
Vitafoam Ltd UK	Memory Foam	Urethane Open-Cell	480 x 480 x 75	0.01728	1.07	63.5	16.1
	Vasco 40						
	MF-75 mm						
Vitafoam Ltd UK	Memory Foam	Urethane Open-Cell	480 x 480 x 100	0.02304	1.46	63.5	16.1
	Vasco 40						
	MF-100 mm						
Vitafoam Ltd UK	Reflex 35 M	Urethane Open-Cell	480 x 480 x 100	0.02304	0.86	37.3	44.9
	Ups-100 mm						
Airex AG	Balance Pads	Polyurethane	440 x 400 x 50	0.0088	0.34	38.6	217.9
Speciality Foams Industrie, Switzerland	BP-50 mm	Closed-cell					

The density was calculated by dividing the mass by the volume. *E* was measured using the data provided by the indentation force deflection test when the specimen was compressed by 25% of its original height.

### Test A: density test

The density of the uncored foam was calculated from the mass and volume of each specimen. The pad’s dimensions (m^3^) were measured with the use of a millimetric measuring tape. The mean mass (kg) was recorded as the average of five measurements with an electronic scale (± 1 g) (Model 1089 BKWHDR, Salter, Hamburg). The density was calculated by the formula:

$$ \mathrm{Density}\kern0.5em =\kern0.5em \mathrm{M}/\mathrm{V} $$

where: M = mass of specimen, kg, and V = volume of specimen, m^3^.

### Test B: indentation force deflection test (IFD)

Based on ASTM standard D-3574-11, this test consisted of measuring the force necessary to produce a predefined indentation in the foam. A flat circular indenter with a 203 mm diameter foot was used to apply a load on the specimen which was supported on a level horizontal plate that was perforated with approximately 6.5 mm holes on approximately 20 mm centres to allow for rapid escape of air during the tests. From the data obtained, the modulus of elasticity was calculated for each specimen with the following formula:

$$ E\kern0.5em =\kern0.5em \frac{\sigma }{\varepsilon}\kern0.5em =\kern0.5em \frac{\frac{F}{A_0}}{\frac{\Delta L}{L_0}}\cdots \mathrm{N}/{\mathrm{m}}^2 $$

where:

- *E* is the Young’s modulus (modulus of elasticity);
- *σ* is the stress applied on the pad;
- *ε* is the strain measured from the application of σ;
- *F* is the force exerted on the foam pad
- *A*_0_ is the original cross-sectional area of the indenter through which the force is applied;
- Δ*L* is the amount by which the height of the pad changes;
- *L*_0_ is the original height of the pad.

### Procedure

The specimen was placed such that the indenter was in the centre of the apparatus’ supporting plate. The area to be tested was preflexed twice by lowering the indenter’s foot to a total deflection of 75% of the full part thickness at a rate of 250 ± 25 mm/min. The specimen was allowed to rest 6 ± 1 min after the preflex. The indenter was then brought into contact with the specimen by applying a 4.5 N load to the indenter’s foot. The specimen was further indented at a rate of 50 ± 5 mm/min to a displacement equal to 25% of the original thickness. The force was then adjusted to retain this displacement for 60 ± 3 s at which point the force measurement was taken. Without unloading the specimen, the deflection was increased to 65% deflection and once more the force was adjusted to retain this displacement for 60 ± 3 s when the force was recorded.

### Test C: modified indentation residual gage length test – specified force (MIRGL)

The traditional “indentation residual gage length” test force (IRGL) used to measure the thickness of the pad under a fixed force of 110 N and 220 N on a 203 mm diameter circular indenter foot [17]. However, these loads were not sufficient to represent the force of an adult standing on the foam pads. For this reason, the ASTM method was modified to use fixed loads of 110 N, 220 N 330 N, 440 N, 550 N, 660 N, 770 N, 880 N, 990 N, 1100 N, 1210 N and 1320 N. Furthermore, we tested the materials with two indenter sizes: 203 mm diameter and 406 mm diameter.

### Procedure

The specimen was preflexed twice with a 330 N force applied at 200 ± 20 mm/min and then allowed to rest after load removal 180 ± 5 sec. Foam pads were tested either as single layer pads (MF: memory foam; Uph: upholstery foam; BP: balance pad) or a combination of two different size bi-layer pads. The first one being a large bi-layer of 0.25 m^2^ surface board (MFL: Memory foam large; UphL: Upholstery foam large; BPL: balance pad large) or with a small bi-layer of 0.09 m^2^ surface board (MFS: Memory foam small; UphS: Upholstery foam small; BPS: balance pad small). The deflection was then recorded after the application of 110 N applied for 60 ± 3 sec. The load was then increased up to 1320 N in steps of 110 N, again holding for 60 ± 3 sec at each load increment. The procedures were repeated a second time with a 406 cm diameter indenter.

### Posturography

Thirty-six healthy male factory workers (mean age = 39.7 years ± 9.3; mass = 88.4 kg ± 14.1; height = 1.78 m ± .034; BMI = 28 ± 3.1) volunteered to participate in this cross-over study. All participants were physically active and none had neurological, vestibular, visual or musculoskeletal complaints at the time of the experimentation. The participants were divided into three groups according to mass (Group 1: less than 60 kg, n = 5; Group 2: 60.1 kg to 89.9 kg, n = 23; Group 3: greater than 90 kg, n = 8). Ethical approval was obtained for the posturography assessment from the University’s Ethics committee and the procedures followed were in accordance with the ethical standards of the Helsinki Declaration of 1975, as revised in 2013 [18]. All participants read the information sheet and signed the consent form.

Postural sway velocity was recorded with the use of a force platform (QPS-200, Midot Medical Technology) linked via a USB connector to a laptop computer and the signal processed with Posture Analyser software (Midot Medical Technology). Postural sway velocities provided by the Posture Analyser software were saved in separate files on a computer.

### Procedure

Posturography was measured three times for each of the 13 randomly-selected experimental situations (no foam, four samples of mono-layered foam, and eight samples of bi-layered foam). The order of each test was determined by a random sequence generator (http://www.random.org/sequences/). The bi-layered form consisted of the foam pad covered by either a square 0.25 m^2^ or 0.09 m^2^ wooden 2 cm thick board. The values of the three posturographic records were averaged and used for analysis.

Participants were instructed to stand on the force platform with their feet together and eyes closed. Recording was started after 30 seconds of quiet stance. After recording was completed, participants were allowed to step off the platform and relax for one minute before the procedure was repeated two additional times. Once the three posturographic recordings were completed, the participants were asked to stand off the force platform and the experimenter changed the foam sample according to the pre-determined sequence. Posturography was again recorded. Sampling was recorded for 30 seconds [19] at 30 Hz per channel.

### Analysis

The overall posturographic data and the participants’ posturographic data grouped by mass were both tested for normality using the Shapiro-Wilk test. Descriptive statistics presented the mean sway velocity ($\overline{x}$)*,* Interquartile range, 95% Confidence Interval for $\overline{x}$ and sway velocity per mass category. Statistical tests were used to determine change in postural sway velocity. One-way repeated measures analyses of variance (ANOVA) with Greenhouse-Geisser corrections were used to compare postural sway velocity in the 13 experimental situations between 1) balance without foam surfaces and 2) with 12 other foam combinations. Wilcoxon-signed rank tests were used to evaluate the difference in sway velocity between mass groups. Levels of significance were set at 0.05 and the Bonferroni post-hoc test was used in the ANOVA and Wilcoxon-signed rank tests. Statistical analyses were performed using SPSS 17.0.

## Results

The density of the tested samples varied from 63.5 kg/m^3^ for the Vitafoam memory foam down to 38.6 kg/m^3^ for the Airex balance pad. Conversely, the memory pads had a value of *E* of 16.1 kPa whilst the balance pad’s *E* was 217.9 kPa (Table 1) when compressed by 25% of their original height.

The indentation force deflection test showed that memory foam pads necessitated much lower loads in order to produce a deflection of 25 and 65% of their original height. Conversely, pads with a larger *E* required a larger force in order to achieve the same deflection as seen in Table 2.

**Table 2:** **Indentation force deflection test (IFD)**

	**203 mm diameter indenter**	
	**Load (N) at 25% thickness reduction**	**Load (N) at 65% thickness reduction**
MF-75 mm	65.2	169.5
MF-100 mm	74.7	200.6
Uph-100 mm	181.3	550.7
BP-50 mm	880.9	4861.2

Force necessary to produce 25% and 65% indentation on four different foam samples. MF-75 mm: memory foam 75 mm thickness; MF-100 mm: memory foam 100 mm; Uph: upholstery foam 100 mm thickness; BP: balance pad 50 mm thickness.

**Figure 2:**
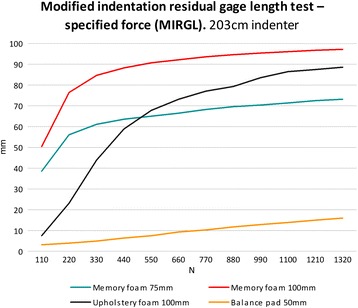
**Results from the modified indentation residual length test using the 203 mm indenter foot.**

**Figure 3:**
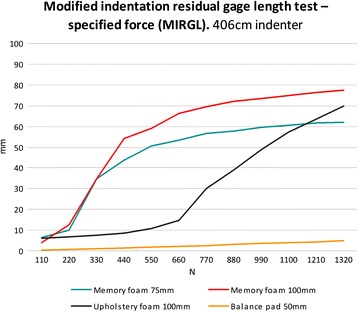
**Results from the modified indentation residual length test using the 406 mm indenter foot.**

The deformations of the foam pads during the MIRGL test using the 203 mm indenter were non-linear with the exception of the balance pad which showed linearity throughout the range of loads applied (Figure 2). Furthermore, both memory foam and upholstery pads were compressed to more than 75% of their original length when a load corresponding to an average male’s weight of 770 N was used (Figure 2). The 406 cm indenter did not alter the memory foam’s linearity during the MIRGL test, in contrast to the upholstery pad which showed a linear deformation from 660 N compression onwards with this larger indenter (Figure 3).

### Posturography

The Shapiro-Wilk normality tests for changes in postural sway velocity in all participants suggested that normality was a reasonable assumption (*p* > 0.05). On the other hand, when participants’ results were stratified by body mass, the velocity data was not normally distributed (*p* < 0.05). The average velocity, coefficient of variation, Interquartile range and average sway velocity according to body mass results according to each foam sample and indenter size are presented in Table 3.

**Table 3:** **Mean, coefficient of variation, interquartile range and confidence intervals for overall posturographic results and participants’ results stratified by body mass**

	$\overline{\mathbf{x}}$ **(SD) mm/s**	**CV**	**IQR**	**95% CI for** $\overline{\mathbf{x}}$		**<60 kg**		**60-89 kg**		**>90 kg**	
						$\overline{\mathbf{x}}$	**Mdn**	$\overline{\mathbf{x}}$	**Mdn**	$\overline{\mathbf{x}}$	**Mdn**
No foam	25.1 (5.2)	0.21	18.7	21.1	27.7	29.1	21	24.0	28.5	24	28.5
MF-75 mm	23.4 (6.1)	0.26	15.5	19.7	22.6	27.1	18	22.3	27.1	21	25.5
MF-100 mm	22.3 (4.9)	0.22	14.7	18.4	21.4	26.1	17	21.2	25.8	20	24.5
Uph-100 mm	75.6 (7.4)	0.10	23.2	69.2	85.7	81.9	82	82.1	49.5	45	49.5
BP-50 mm	78.7 (7.1)	0.09	23.7	74.1	82.7	83.2	79	82.0	66.5	62	66.5
MFL-75 mm	27.0 (4.6)	0.17	18.3	23.1	27.7	30.9	24	26.1	28.8	24.3	28.8
MFL-100 mm	32.8 (6.9)	0.21	18.7	28.8	29.7	36.8	36	31.4	30.5	26	30.5
UphL-100 mm	64.5 (6.3)	0.10	24.5	59.3	67.7	69.7	64	57.0	81.4	76	81.4
BPL-50 mm	33.0 (2.5)	0.08	19.75	29.2	33.7	36.9	30	33.0	32.5	28	32.5
MFS-75 mm	25.5 (6.6)	0,25	19.1	21.4	26.8	29.6	21.4	24.4	28.7	24.1	28.6
MFS-100 mm	29.0 (5.3)	0.18	18.5	24.5	42.7	33.4	39	24.6	28.6	23.5	28.0
UphS-100 mm	48.4 (3.7)	0.07	20.5	43.6	44.7	53.3	41	44.0	63.5	59	63.5
BPS-50 mm	34.0 (3.1)	0.09	19.8	30.0	34.7	38.1	31	34.0	33.5	29	33.5

$\overline{x}$ (SD) = average velocity and its standard deviation. IQR = Interquartile range. CI – 95% confidence interval for the average velocity of sway.

MF: memory foam; Uph: upholstery foam; BP: balance pad; Large bi-layer with a surface of 0. 25 m^2^ board: MFL: Memory foam large; UphL: Upholstery foam large; BPL: balance pad large; Small bi-layer with a surface of 0.09 m^2^: MFS: Memory foam small; UphS: Upholstery foam small; BPS: balance pad small.

A repeated measures ANOVA with a Greenhouse-Geisser correction determined that postural sway velocity differed significantly between surfaces measured (F(1.984, 22.257) = 21926.764, P < 0.0001). Post hoc tests using the Bonferroni correction revealed that postural sway velocity was significantly increased especially when standing on a monolayer upholstery foam and on a monolayer balance pad (75.6 ± .18.7 mm/s and 78.7 ± 13.5 mm/s respectively) (Table 4).

**Table 4:** **ANOVA Pairwise comparison between velocity of sway without foam and with different foam surfaces**

		***p***	**95% CI for difference with no foam**	
Mono-layer	MF-75 mm	ns	−0.3	3.7
	MF-100 mm	.002	0.6	5.0
	Uph-100 mm	.000	−61.0	−39.9
	BP-50 mm	.000	−58.8	−48.2
Bi-layer 0.09 m^2^	MFL-75 mm	.030	−3.6	−0.08
	MFL-100 mm	ns	−15.1	−0.2
	UphL-100 mm	.000	−44.5	−34.1
	BPL-50Lmm	.000	−9.2	−6.5
Bi-layer 0.25 m^2^	MFS-75 mm	ns	−2.2	0.8
	MFS-100 mm	ns	−8.4	0.6
	UphS-100 mm	.000	−27.2	−19.3
	BPS-50 mm	.000	−10.2	−7.5

ns = not significant.

Wilcoxon signed-rank tests with Bonferroni corrections showed that in three experimental situations, the postural sway velocities were significantly different in participants of different masses (<60 kg vs 60-89 kg, upholstery foam, Z = −6.156, p = .009; 60 kg vs >90 kg, upholstery foam, Z = −1.950, p = .012; <60 kg vs 60-89 kg, upholstery foam and large board, Z = −2.646, p = .010; 60-89 kg vs >90 kg, upholstery foam and large board, Z = −2.521, p = .012).

## Discussion

The objective of this project was to determine which type of foam pads were the most effective to enhance postural disturbances according to participant’s weight.

### Foam pads

Balance during quiet stance has been shown to be a good representation of overall system health, but not a good measure of underlying pathophysiology due to numerous contributing factors potentially affecting balance. Static posturography can be altered in different ways in order to challenge the participants to maintain a stable posture for example, narrowing the base of support by having the feet close to each other, decreasing visual feedback (closing eyes), altering the standing surface to decrease proprioceptive feedback, or introducing an accessory task or action during balance recording [20]. An increased average centre of pressure change has been associated with aging, obesity, neuropathy, Parkinson’s disease, vestibular loss, stroke etc. [7,21-23]. The usefulness in using foam pads to decrease cutaneous plantar proprioception during posturographic measurement is fairly well established [11,24-26]. However, the types of foam pads used in previous experimentation has differed and it is difficult to compare results between studies. Through compression testing of different types of open cell foams using ASTM standard D-3574-95, our study has shown that stiffness varies as the material specimen cross-section changes in size relative to the indenter. We have demonstrated that foams pads, with the exception of the balance pad and the upholstery foam with the 406 mm indenter, did not show linear deformation throughout the range of loads used in the MIRGL with both the 203 mm and 406 mm indenters. Both memory foam pads failed to resist the compression at relatively low loads which suggested they would not provide sufficient resistance to compression during posturography for healthy adult participants. Their compression slopes during the MIRGL clearly show a trend towards asymptotic displacement beyond 220 N for the memory foam and beyond 660 N compression for the mono-layer upholstery foam. Non-linear stress–strain relationships were observed due to the changes in the foam geometry at high strains. When the foam is highly compressed the foam volume tends to zero and the stiffness tends to infinity. Patel suggested that such large compression (as observed with the memory foam here) would result in the participants coming in to close contract with the rigid surface beneath the foam [13]. The balance pad showed a largely linear response throughout the loads applied during the MIRGL test. Conversely, the upholstery foam exhibited a bi-linear type of behaviour when compressed with the 406 mm diameter indenter. It supported the load with minimum deformation up to 660 N, at which point it gave way and deformed with a lower stiffness up to the maximum load.

Our results show that foam pads can indeed increase the postural sway velocity of healthy participants, in some cases significantly. Participants standing on foam pads did elevate their centre of mass by nearly 50 mm corresponding to less than 3% of the participants’ average height. The force platform used in this project consisted of 4 weighing plates, and the CoP is calculated from the resultant force, with the velocity calculated by the change in the CoP position. It is therefore unlikely that elevating the centre of mass would have affected appreciably the postural sway velocity results. When participant data were stratified according to mass, results showed that the balance pad did still produce the largest increase in sway velocity in Groups 1 and 2, in the heavier Group 3 (mass > 90 kg), the large bi-layer upholstery foam pad had the largest effect. The pairwise comparison between sway velocities without foam and with foam surfaces showed a large confidence interval, which can be attributed to the separation of participants into smaller groups according to mass. The null hypothesis stating “there is no difference in sway velocity when any of the foam pads are used” can thus be rejected. Furthermore, our participants were male factory workers with a mean BMI of 28 which is slightly higher than the UK male average [27]. Athletes presenting the same mass but with a more mesomorphic body type might have provided different results.

It is interesting to note that not only was there no significant difference between posturographic results between “no foam” and samples of 75 mm and 100 mm thick memory foam samples, but the sway velocity was somewhat improved when memory foam pad were used. A learning effect explanation can be excluded in view of the random order of the test conducted. We concluded that because the participants’ feet had a smaller cross-sectional area than the pads onto which they were standing on and the fact that our participants nearly flattened the pads meant a shear force was created between the material and the sides of the feet as the specimen deformed. This would have increased the surface area of contact between the foam pads and the side of the feet which in turn would most likely have increased proprioception thereby providing additional cues and improving balance. With participants of larger mass, as the deflection increased, the memory foam could actually have provided an advantage in the posturographic task. Thus, when selecting a type of foam pad to be used in posturography, it is recommended that investigators select samples appropriate to their participants’ weight. For instance, in the selection of foam pads for individuals weighting more than 900 N, a bi-layer upholstery foam pad of around 37.3 kPa and 44.9 kPa such as used in our experiment would be the appropriate choice. Additionally, it may be of importance to select a foam pad presenting limited deflection under loading in order to avoid contact of the feet with the sides of the material.

## Conclusion

The Balance pad produced the largest postural sway velocity in participants with less than 90 kg mass whilst the bi-layer upholstery sample (406 mm indenter) produced the largest changes in participants above 90 kg of mass. The results suggest that foam pads selected for static computerised posturography: 1) could possess a modulus of elasticity of around 40 kg/m^3^, and 2) show linear deformation properties matched to the participants’ weight.
